# Gender and kidney transplantation

**DOI:** 10.3389/fneph.2024.1360856

**Published:** 2024-04-22

**Authors:** Arushi Nautiyal, Soumita Bagchi, Shyam Bihari Bansal

**Affiliations:** ^1^Department of Nephrology, Jaipur Golden Hospital, New Delhi, India; ^2^Department of Nephrology, All India Institute of Medical Sciences, New Delhi, India; ^3^Department of Nephrology, Medanta - Medicity, Gurugram, India

**Keywords:** gender, kidney, transplantation, sex, women, India

## Abstract

Kidney transplantation provides the best form of kidney replacement therapy with improvement in quality of life and longevity. However, disparity exists in its availability, utilisation and outcomes, not only due to donor availability or financial constraints but also arising from the influence of biological sex and its sociocultural attribute i.e., Gender. Women make up the majority of kidney donors but are less likely to be counselled regarding transpantation, be waitlisted or receive living/deceased donor kidney. Biological differences also contribute to differences in kidney transplantation among the sexes. Women are more likely to be sensitised owing to pregnancy, especially in multiparous individuals, complicating donor compatibility. A heightened immune system in women, evidenced by more autoimmune illnesses, increases the risk of allograft rejection and loss. Differences in the pharmacokinetics of transplant drugs owing to biological variances could also contribute to variability in outcomes. Transgender medicine is also increasingly becoming a relevant topic of study, providing greater challenges in the form of hormonal manipulations and anatomic changes. It is thus important to determine and study transplantation and its nuances in this backdrop to be able to provide relevant sex and gender-specific interventions and design better practices for optimum kidney transplant utilisation and outcomes.

## 1 Introduction

The world is becoming increasingly inclusive and plural with pushback on discrimination of any kind. However, a basic sex and gender-based disparity remains everywhere including in healthcare. This exists at all levels encompassing access to healthcare, its utilisation and outcomes as well as research. Some of this disparity arises from the biological differences related to sex, whereas the rest stems from its behavioural and sociocultural attributes i.e., gender (1). Biological variances between the sexes at the genetic, hormonal and anatomic levels may alter disease phenotypes; similarly, behavioural traits and cultural factors relating to gender can modify disease perception, treatment-seeking behaviour and coping mechanisms. Therefore, the epidemiology, course, response to treatment and outcome of many diseases may vary depending on sex and gender.

Previously, human and animal model-based research would pool data or extrapolate from the commonly male majority towards the rest of the population (1, 2). Steinberg et al, in a cross-sectional analysis of 20,000 plus studies over recent two decades, were able to identify increased reporting of sex-based data, but also found a sex-based bias in research enrolment in various disciplines, often determined by the primary purpose of the study; the female sex was found to be under-represented in nephrology and genitourinary studies (3). Research governing bodies and scientific journals are now increasingly mandating sex and gender-based analysis in both clinical and pre-clinical settings. Adoption of such practices would allow for the discovery of clinically relevant dissimilarities to allow targeted therapeutic interventions (1).

In the realm of kidney disorders, chronic kidney disease (CKD) is a growing public concern associated with notable morbidity and mortality, especially with the projection rates of diabetes set to rise significantly; as diabetes is the most common cause of kidney dysfunction (4, 5). Worldwide, the majority of population studies report pre-dialysis CKD prevalence rates to be higher in women than men except for a few populations (6). However, in India, women have been consistently under-represented in population data studies focussing on CKD; in some large studies, they only comprised one-third of the study population (5, 7, 8). The authors do comment on possible sociocultural factors affecting health-seeking behaviour in females in low and middle-income countries like India (5).

The prevalence of common causes of CKD like diabetes, hypertension, and chronic interstitial nephritis are similar in men and women (5). However, autoimmune diseases like systemic lupus nephritis are more common in women. Pregnancy is another situation, exposing women to hypertensive disorders, acute kidney injury (AKI), complement dysfunction, and worsening of CKD if present antenatally (6, 9, 10).

Despite higher numbers in the prevalent CKD population, various studies have shown a lesser number of women progress to end-stage kidney disease (ESKD) in comparison to men. Multiple hypotheses have emerged to explain the lower rate of CKD progression among women including incorrect eGFR-based calibration, kidney protective effects of the female hormonal milieu and deleterious effect of testosterone demonstrated in experimental studies (11) and higher prevalence of unhealthy lifestyles among men. There is also a gender-based discrepancy in dialysis initiation rates. A knowledge gap exists among women about their disease and treatment options and they more often tend to opt for conservative management or defer dialysis, especially when elderly (11, 12). There is also a disparity in the access to chronic disease care and kidney replacement therapies(KRT) among men and women and this bias may vary across geographies being more prominent in low and middle-income countries(LMICs) like India compared to high-income countries in the West.

### 1.1 Gender and access to kidney transplantation

Kidney transplantation (KT) provides the best modality of kidney replacement therapy (KRT), it is associated with a survival benefit and lesser morbidity than dialysis in any form (13). It also allows for a close to normal resumption of day-to-day living, thus improving quality of life and is cost-effective in the long term as compared to dialysis (14).

However, a disparity exists in kidney transplantation too. In USRDS data, the rates of waitlisting and subsequent transplantation for women continue to be lower than men, in both deceased donor as well as living donor transplantation. In 2020, rates for kidney transplants among women were 3.5 compared to 4 per 100 person-years for men in the US (15). In low and -middle-income countries like India, data is often difficult to come by, often represented by single-centre or regional studies (16, 17). In a single centre report from north India, only 11.1% transplant recipients were women. 66.1% kidney donors were women with 90.7% of spousal donors being wives (16). In a large public sector transplant hospital in Gujarat, India, KT rates in women were close to one-fifth of those of men (17).

Women are less likely to be counselled regarding kidney transplantation by their healthcare providers (18, 19), though cardiovascular morbidities are more common in men than women in the ESKD population (12, 20, 21). Segev et al, conducted a USRDS-based registry data study spanning 5 years, they were able to identify that with increasing age and co-morbidities, women had less access to transplants compared to men with similar profiles; though the survival benefit of transplants was similar. This disparity was attributed to perceived frailness by physicians, patients or family members (19). A recent multi-regional cohort study from USA observed interaction between gender, age and race with regard to kidney transplant referral, found that older non-Hispanic black and white women were less likely to be referred for a transplant compared to men (22),.

In a multicentric, cross-sectional survey of outpatients at dialysis centres, Salter and colleagues, found that older adults and women of all ages had fewer discussions with both healthcare providers and their social groups regarding KT (23). A lack of information and awareness regarding treatment options is the first hurdle in identifying an optimum therapeutic plan for oneself, and thus the role of healthcare providers in educating their patients, especially women is paramount. In a retrospective analysis, Monson et al, found white and black women to have slower rates of completion of pre-transplant medical evaluation in comparison to white and Hispanic men (24).

It is imperative to have social support in navigating KT, in identifying a living donor if possible as well as completing the investigation process and follow-up. Women are often the primary caregivers in a family unit (25). Studies have shown a differential level of care received by older women suffering from disability than men (18, 26), such experiences may lead to apprehensions among women regarding social support available for their care in the peri-operative period.

In a situation with multiple co-morbidities, self-advocacy and patient enthusiasm are often a catalyst for transplant consideration; however, in studies on access to transplant information, women are less inclined to accept counselling regarding KT (27, 28), expressing more health-related and psychological concerns regarding transplantation than their male counterparts (21, 29). Frailty and personal perceptions regarding the same can lead to concerns regarding the ability to withstand surgical stress and immunosuppression, perhaps leading to hesitation in contemplating transplantation both by the patient and their healthcare provider. Adoption of objective measures of frailty by healthcare providers, such as the Fried physical frailty phenotype, as opposed to subjective screening and targeted interventions where possible, could help improve access to transplants for women and the elderly (30). This is especially important as studies have shown an improvement in frailty scores (31) and survival benefit post-transplantation in all groups (19). Educational programmes and the involvement of social support groups may help women address their apprehensions about transplantation. Depression and behavioural disorders are more prevalent in women in the ESKD population (12, 21), involvement of mental health specialists, counselling and therapy could further help in the acceptance of transplantation as a preferred modality for KRT among women. Factors affecting access to transplantation and suggestions to improve outcomes are listed in **Table 1**.

**Table 1:** Factors affecting renal transplant access and measures to improve outcomes.

Factors Affecting Transplant Access	Suggested Measures to improve outcomes
Community awareness of kidney disease and treatment options	Improved Government policies, NGOs, training of local healthcare workers, skits and educational programmes in local languages
Economic constraints	State based health schemes, rural and urban-poor insurance schemes, NGOs
Change in Gender based attitudes and norms/Familial roles	Increasing literacy rates and skill development especially for females; increasing share of females in workforce, community sensitisation, Advocacy groups
Medical issues impeding KT	Patient education campaigns regarding diet, exercise, avoidance of smoking, blood pressure and blood glucose management; improving frailty indices; optimising medications; educational information leaflets; patient help groups
Gender disparities in kidney donation	Empowering women, financial independence, increasing literacy in women, Donor advocacy
Health worker bias	Review local and state wise data. Introspection of practises. Independent donor advocate. Encourage shared decision making. Measures to increase community awareness
Improving transplant outcomes	Counselling regarding need for medication adherence especially among adolescents, follow up visits. Education regarding and ensuring clean water supply, maintenance of sanitation. Navigating contraception and pregnancy. Research into optimising individual immunosuppression, precision medicine. Emphasising on sex and gender based analysis in research.

NG0, non-government organization; KT, kidney transplantation.

### 1.2 Gender and access to pediatric transplantation

Studies in pediatric KT have also shown a gender disparity; A European study involving 35 countries found that although overall transplant rates were similar among boys and girls, pre-emptive transplants were lower in girls by 23% in comparison to boys, leading to longer times spent on dialysis (32). This was not explained by medical factors alone and parental and healthcare provider attitudes or bias was considered as probable cause. Girls were also found to be waitlisted less than boys for deceased donor transplants (33).

### 1.3 Social, economic factors generating gender bias in access to healthcare and kidney transplantation

The financial burden of transplantation is an important consideration as KT is associated with high initial costs (direct and indirect) but the overall cost is less in long-term as compared to those on dialysis (34). Even in countries with universal healthcare like the United Kingdom, lower transplantation rates have been reported in socially deprived subgroups (defined by unemployment, car ownership, home ownership, and overcrowding) (35). Couchoud et al, also found older, non-working women in France to be less likely to be waitlisted for KT (21).

As the majority of healthcare expenditure in India tends to be out of pocket, chronic medical illness often places severe financial constraints on families. Gender disparity in all aspects of healthcare expenditure has been widely documented in India (36), which may play a huge role in inequity in kidney transplantation. Men are usually the primary breadwinner in the family and are therefore more likely to be prioritized for financial and social support for KRT than women. Women are often unemployed and financially dependent, assuming non-paying household work and caregiving duties (37). Women in India have lower health literacy with lesser access to communication media (37). In patriarchal societies, such as India, women also tend to have less agency for themselves, even in aspects about healthcare and lack independence to take treatment decisions which are often made by their male family members. Similar gender based inequalities have been noted among other LMICs (38). Even in high income countries where health awareness among women is considered to be greater, with studies even reporting higher primary care utilisation by women perceived unmet health needs were found to be higher among women than men (39), gender differences in critical care have also been described with women receiving less invasive therapeutic interventions (40).

Depression and anxiety disorders are also more common in women, often fuelled by poverty (41), making them susceptible to defer initiation of KRT, let alone transplantation; mortality in such patients is often unaccounted for (12). A qualitative study exploring nephrologists’ perspectives highlighted gender stereotyping, stigma and prejudice with men being vested with decision making powers and educational and financial handicaps being the major factors contributing to the gender disparities in access to KT (42). These social factors were considered to be significant even though most of the nephrologists interviewed were from high income developed countries(Australia, USA and Austria) and likely to be of greater concern in traditional societies and LMICs.

Increasing education and awareness in the general population and challenging traditional gender roles in communities could bring improvement in access to healthcare. Recognising and changing restrictive gender norms as well as impugning practises that maintain them in communities at the grass root level is required through social and economic policies. Similarly a change in gender-based attitudes among healthcare providers is required to remove biases and improve both primary and specialty care and community health.

Significant barriers in transplantation and CKD outcomes are also seen based on a rural-urban divide, which may also add to gender based disparity. Pertinent factors such as distance from available healthcare facilities, quality of nearest facilities, laboratory and imaging services available weigh appreciably on community health and KT (43, 44). Considerable distance from adequate healthcare facilities and poor transport infrastructure affects health seeking behaviour (44, 45) and may make medication availability a considerable challenge. Linguistic barriers, inability or frustration in navigating healthcare systems, need for geographic relocation and ensuing economic costs add to the challenges for kidney replacement therapies including transplant for rural communities, Ensuring clean water supply and ability to maintain sanitation and hygiene can be a task in underserved rural and urban poor dwellings, increasing risks of infections.

Policies for rural healthcare, transport and clean water access need to be strengthened and regularly reassessed by local governments and stakeholders, increasing use of telehealth and remote monitoring can allow for better follow up, local health auxiliary workers can help co-ordinate such communication with specialists and overcome language barriers.

### 1.4 Medical issues, gender and access to kidney transplantation

Even following waitlisting, women are less likely to receive deceased donor transplants (6, 20). Various studies including donors from any source, have found a higher body mass index (46), type 2 diabetes mellitus causing CKD (20), and higher panel reactive antibodies (18) among others as reasons for such disparity. Obesity predisposes to more surgical risk; women have greater body fat percentage than men which may lead to physician-centred bias in proceeding with transplantation (46). In a retrospective analysis of USRDS data, focussing on differential deceased donor transplantation rates among the sexes, based on the cause of CKD, Ahearn and colleagues found that women with type 2 diabetes mellitus, were less likely to receive KT, despite having lesser cardiovascular comorbidity than their male counterparts with diabetes (20). Pregnancy and subsequent sensitisation, leading to HLA incompatibilities, especially with spouses and children as potential donors creates barriers for women in living donor transplantation. Studies have also reported the differential sensitisation to be largely contributed by pregnancy more than other sensitising events such as blood transfusion or previous transplant (47, 48).

## 2 Gender and kidney donation

The majority of living kidney donors tend to be female. In India, living kidney donation constitutes the bulk of kidney transplantation in the country (17, 49–51). These trends have also been reported in other countries like China (52) and Turkey (53). Kurnikowski et al, conducted an analysis of sex distribution of donors based on varying sources of data from multiple countries; it found that in the majority of the sampled countries, female donors outnumbered males and the donation rates were disproportionate to their representation in the general population. Similarly, females were less likely to be transplant recipients than males. The authors hypothesised that reduced tobacco use among women and overall lower employment rate among females increase their availability as donors (54). A study in a single large public transplant centre in India found female predominance among kidney donors in all categories, whether parental, spousal or sibling (17). In recent decades, spousal donation rates have been increasing steadily; shrinking family units may be responsible for such trends and these include predominantly female donors (49). Zimmermann et al., in an analysis of potential donor pools for transplant recipients in Canada, also found greater female predominance among donors, fuelled mostly by spousal donation (55). However a change in such trends in recent times has been noted (56). Biological factors responsible for lesser donation among men include an immunological barrier in husband to wife donation, unhealthy lifestyle choices or population-based, evidence of greater hypertension, and heart disease among men (54, 57). In a registry-based analysis of donor safety, though absolute numbers were low, men also had significantly greater perioperative mortality than women (58). However, social factors have been considered to contribute more towards the lesser number of male donors (59).

A gender difference in attitudes towards organ donation has been seen in community studies. Women are traditionally perceived as caregivers and hence more forthcoming for donation, based on greater empathy and altruistic tendencies. Almeida et al, in a general population-based survey, found females to have a more positive attitude towards kidney donation in comparison to men (60). Similarly, in a survey of adults in the United States, Yee and colleagues found that women were more willing to donate organs to family members and strangers than men (61). An improved quality of life of the partner and lesser caregiving requirements in opting for kidney transplants over other forms of kidney replacement therapy for spousal donors are also frequent considerations, especially for women who tend to shoulder the bulk of caregiving duties (62). Living kidney transplantation can often put an emotional and economic strain in the family (63); fears about adverse consequences and lost income underlies an unwillingness to involve family members with more earning potential in the donation process, more likely to be male. Coercion and manipulation from family members also influence decisions for donation, especially in the case of female donors who are often uneducated and unemployed (37, 60), and there should be safeguards to prevent this during the donor review process.

### 2.1 A survey on gender discrimination and kidney transplantation in India:

**Figure 1:**
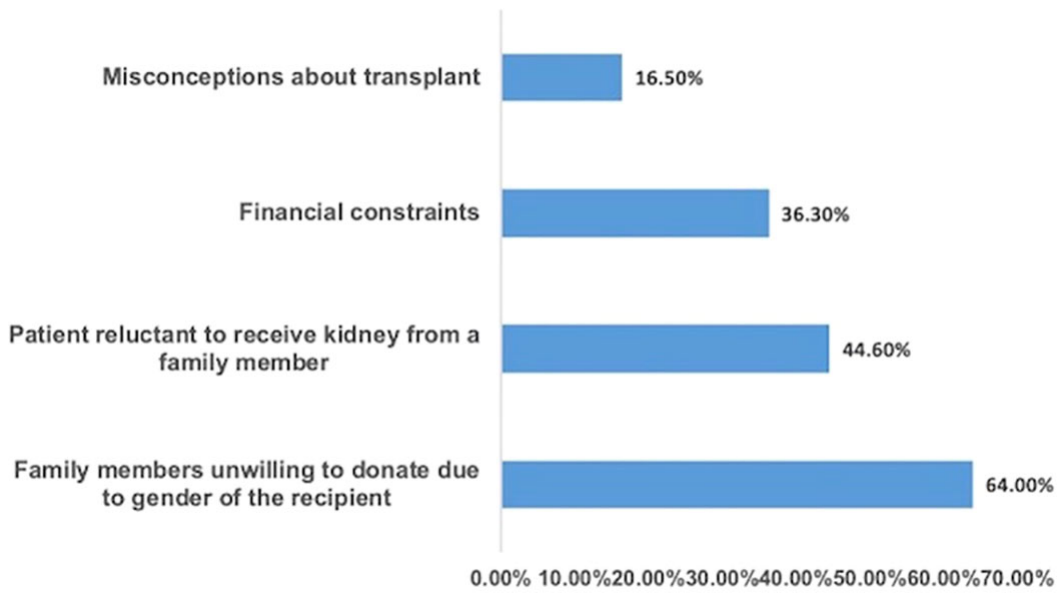
Reasons attributed by nephrologists for gender disparity in access to transplantation.

We recently conducted a survey among Indian Nephrologists regarding access to kidney transplants based on gender (Unpublished). There were 267 respondents, and 80% answered that in their practice women comprise < 25% of recipients and > 75% of donors. Women were less likely to receive KRT or KT as compared to males. About the reasons for this disparity in getting KT: 16.5% cited the misconception about transplant, 36% financial reason, 44% said that women were reluctant to take a kidney from a family member and 64% responded that the family members were unwilling to donate due to female gender. The survey also found out that in the case of women being recipients, 85% of donors were parents, however, if a male was a recipient, then approximately 70% of donors were spouses (**Figure 1**).

## 3 Gender and transplant outcomes:

Kidney transplant outcomes are affected by various factors including immune activity, medication compliance, donor characteristics, and dialysis vintage among others, some of which are influenced by sex and gender aspects. When assessing for differences in transplant outcomes according to sex, various studies have shown conflicting data with some showing poorer results for female recipients while others have revealed no long-term differences (63–66). Females are also more likely to react to the sex- dependant H-Y minor histocompatibility antigens found in male donor kidneys (67).

In a retrospective analysis of deceased donor recipients using the Scientific Registry of Transplant Recipients (SRTR) database, Lepetyre et al, studied the interaction between donor sex, recipient age and recipient sex with graft outcomes; they found that females of all ages had poorer graft outcomes when receiving male kidneys, whereas only females between the ages of 15-24 years did poorly even with female donors compared to male recipients (68). In comparison, Vinson et al, in a multinational analysis using SRTR, Australia and New Zealand Dialysis and Transplant Registry, and Collaborative Transplant Study registry data, which included countries providing universal healthcare, identified higher graft loss in young females receiving male kidneys (likely from H-Y antigen effect), they also found lower graft survival in older males recipients than females receiving female kidney, which was considered to be associated with better medication adherence in females (69). Mortality risk is often linked intricately with the risk of graft loss in transplant patients. In an analysis of sex differences in excess mortality (greater than general population mortality rates for that sex) among recipients of a first deceased donor kidney transplant belonging to three large international transplant databases. Excess mortality in female recipients (except ages 45-59 years) was identified, more in the younger than older members of the cohorts, being statistically significant only if the donor was male. Mortality in younger women was more due to loss of graft function, whereas in older women more deaths with functioning grafts were reported (70). Some of the important studies regarding gender and Transplant are shown in **Table 2**.

**Table 2:** The important studies on gender disparity in transplantation.

Authors (Country)	Type of Study	AIms	Results
**Bal (** 16**) (Kerela, India)**	Single centre, Retrospective analysis of LDKT, 2001-2005	To study the gender disparity in LDKT	682 LDKT recipients 88.9% male, 11.1% female, Donors- 66.1%- female 33.9% male
**Segev (** 19**) (United States)**	National Cohort study, USRDS, 2000-2005	Study points- Gender disparities- • Access to transplant (ATT) • Survival benefit after Transplant (SBT)	Multivariate analysis - Overall women had 11% less ATT Increasing significantly for age and comorbidities - more than 75 yr olds- 59% less ATT- likely perception of frailty No difference in SBT between men and women of all ages, irrespective of co-morbidities
**Gill (** 46) (2014) **Bromberger** (47) (2017)	Retrospective, USRDS, Incident ESKD pts (1995-2007) Single centre, intention to treat analysis, Prospective cohort study, Pennsylvania, US 2587 LDKT candidates 62% male, 38% female	Aim- determine association of BMI with access to KT (deceased and living donor) in men and women To identify sex specific points of attrition in LDKT process	Females- BMI>25 kg/m2 -lower likelihood of transplantation from any donor source (HR, 0.75; [95% CI], 0.73 to 0.77) Male- BMI >40kg/m^2^ – lower likelihood of living donor BMI>35kg/m^2^ – lower deceased donor Tx -Similar referrals -Similar rates of crossmatch -Among crossmatched candidates- 50% men vs 35% women received LDKT (p-0.01) -31% potential donor loss in females vs 9% for males - cPRA men =7% ± 22% versus women =24% ± 35%; *P*<0.001) - living donor incompatibility -significantly higher than predicted by cPRA among weakly sensitized candidates with a history of pregnancy
**Vinson (** 69**) (2022)**	Retrospective cohort study • American SRTR • Australia and New Zealand Dialysis and Transplant (ANZDATA) Registry • International Collaborative Transplant Study (CTS) database	Aim- compare graft loss rates between male and female recipients accounting for the modifying effects of recipient age and donor sex	**Male donor-** More graft loss in young females • 0–12 y: adjusted hazard ratio [aHR] 1.42, (95% [CI], 1.17-1.73) • 13–24 y: 1.24 (1.17-1.32) • 25–44 y: 1.09 (1.06-1.13) (likely HY effect) **Female donor-** More graft loss in older males in comparison to older females • aHR 0.93 (0.89–0.98) in 45–59 y-old • 0.89 (0.86–0.93) in ≥ 60 y-old recipients (likely better medication adherence in females)
**Vinson (** 70**) (2023)**	Retrospective cohort study of first deceased donor KT • American SRTR • ANZDATA • CTS	Aim- compare **excess risk of mortality** by recipient sex (excess in comparison to sex and age matched general population)	**Male donor-** Female recipients 0–12 years (Relative Excess Risk 1.54, 95% CI 1.20–1.99) 13–24 years (1.17, 1.01–1.34) 25–44 years (1.11, 1.05–1.18) > 60 years (1.05, 1.02– 1.08) showed higher excess mortality risks than male recipients of the same age except for 45-60 years (only for male donors) Younger females mortality associated with graft loss Older females more death with functioning grafts

LDKT, Lliving donor kidney transplantation; USRDS, United states renal data system; ESKD, End stage kidney disease; BMI, Body mass index; KT, Kidney transplantation; cPRA, Calculated panel reactive antibody; SRTR, Scientific registry of transplant recipients.

Innate and adaptive immunity differs by sex and age. Post pubertal effect of sex steroids on immunity leads to a more robust response in women under the influence of estrogen, whereas androgens have been found to have immunosuppressive effects. This dimorphism predisposes men to infections and women to enhanced immune reactivity which may be harmful in certain situations and be responsible for a greater amount of autoimmune diseases in women as well. In the post-menopausal state, women tend to experience a rapid decline in sex hormone effect which may diminish immune responses (66, 71). This difference may explain the greater likelihood of graft failure in younger women (68, 69). Greater reactivity to male donor kidneys in female recipients likely stems from differences in sexually determined alloantigens such as H-Y minor histocompatibility antigens. The immunosenescence that develops with age, may reduce the reactivity in older female recipients to H-Y antigens. These antigens, expressed by the Y chromosome, are present in all male tissues (65, 68). Their expression varies in different tissues and is prognostically found to be most important in stem cell transplants. However, even in the setting of KT, they have been found to have a significant influence, especially in the short term; Tan et al, demonstrated antibodies to H-Y antigens in female recipients of male kidneys which showed a strong association with acute rejection in multivariate analysis (67).

Medication adherence has been frequently reported to be greater in females (72, 73). Donor recipient weight mismatch (74) with women usually weighing less, leading to nephron underdosing and sex-related differences in metabolic demand on the graft which tend to be higher in men (75) have also been considered as non-immunological factors responsible for graft dysfunction.

Cancer risk in kidney transplant recipients has been greater than in the general population, increasing with transplant vintage (76). Studies looking at gender differences in cancer incidences have found conflicting data, with some observing greater risk in males (77) whereas others reported higher risk in females (76). Webster et al, assessed cancer risk among the sexes stratified by age and found greater risk in younger women than men transplanted at the same age, similar rates in middle-aged recipients and greater rates in older men (78).

## 4 Effect of gender on transplant immunosuppression

Immunosuppression is the backbone of organ transplantation, the most commonly used medications for maintenance immunosuppression in KT include- Calcineurin inhibitors (CNI) i.e. Cyclosporine and the more commonly used drug Tacrolimus (TAC), antimetabolites- such as Mycophenolate mofetil (MMF) and Azathioprine, and steroids. Lifelong intake of these medications is necessary for the long-term survival of graft, however, these drugs are associated with many adverse effects including increased risk of infections due to lowered immunity, drug and food interactions and long-term consequences such as malignancy. CNIs are drugs of a narrow therapeutic index, necessitating drug-level monitoring, thus ascertaining the optimum dose for any individual is of utmost importance. Sex and gender-related variations in drug metabolism have been noted and a greater understanding of the differing pharmacokinetics and pharmacodynamics would help in individualising immunosuppressive regimens.

CNIs are metabolised by the CYP3A4/5 subfamily of enzymes and are substrates of efflux transporter p-glycoprotein; polymorphisms in genes encoding these proteins and other factors such as diarrhoea and drug and food interactions are responsible for the great amount of inter and intra-individual variability in CNI drug levels (79). Tornatore et al. found greater cumulative, neurological and aesthetic adverse effects from tacrolimus in women than men, especially black women (80). Gender and race-related differences have been found in various studies assessing the pharmacokinetics and pharmacodynamics CNIs, but the results have not been conclusive and thus sex specified doses are as yet not recommended (79).

In a pharmacokinetic study of MPA, Morissette et al, found significantly higher ratios of MPA metabolite to MPA in men than women, showing higher clearance in men (81). Sex hormones have been documented to modulate the metabolism of MPA (79). Azathioprine is another antimetabolite which is often used in situations where mycophenolate is not tolerated or in pregnancy. Its converted to its active metabolite- 6-mercaptopurine, which is metabolised by thiopurine S-methyl transferase enzyme (TPMT). The inactivating enzyme expression has been found to be higher in men and influenced by testosterone, however, the clinical implications of this difference are still not clear (82).

Glucocorticoids are a common part of triple-drug immunosuppression regimens for kidney transplantation. Prednisolone has been found to have reduced rates of clearance in women in comparison to men, and thus increased systemic exposure. However, Magee et al. also found that in addition to reduced clearance rates, women have a greater volume of distribution of prednisolone which leads to similar half-lives in both sexes (83). Clearance of unbound prednisolone has also been found to be lower in post-menopausal women as opposed to premenopausal women with no effect of hormone replacement therapies (84). Glucocorticoids are associated with significant adverse effects and thus efforts to streamline optimal doses to reduce steroid exposure could be helpful and need further study.

MTOR inhibitors such as Sirolimus and its derivative Everolimus are also used in alternate immunosuppression regimens. They are also substrates of CYP3A and p-glycoprotein. Sirolimus clearance has been found to be higher in females by 20% (sirolimus), however, no major pharmacokinetic differences by sex have been documented with everolimus (79).

## 5 Kidney transplantation in the transgender population

Transgender refers to individuals whose gender identity does not conform to the sex they were assigned at birth. A substantial proportion of such persons express a desire to transition to the gender they identify with, involving hormonal manipulations and often gender-affirming surgeries. Kidney transplantation in such populations is associated with some unique challenges involving use of hormonal therapy, anatomic changes from gender-affirming surgeries, and psychosocial issues among others (18).

Some trans-individuals opt for gender-affirming surgeries, which may include mastectomy, breast augmentation, facial surgery or urogenital surgeries such as phalloplasty or vaginoplasty. Gender-affirming surgeries have been shown to reduce gender dysphoria. This is a newly advancing field with implications in kidney transplantation as urogenital surgeries with manipulation of urethra can result in strictures, and fistulas or lead to recurrent urinary tract infections (85, 86). Thus medical providers should inquire about and discuss intentions for gender-affirming surgery with transgender patients both prior to and after kidney transplantation (85).

Hormonal therapy can have medical and surgical implications in KT. Feminising medications often include estrogen as oral, transdermal gel or intramuscular preparations. Ethinyl estradiol has been known to increase the risk of venous thromboembolism and is generally held for 2-4 weeks before and after the operative procedure, though such discontinuation may lead to dysphoria (18, 85). Estrogen has also been found to increase tacrolimus levels which may necessitate dose reductions (87), but it requires more studies. Antiandrogens like spironolactone may lead to hyperkalemia which can interact with concomitant transplant drugs like Calcineurin inhibitors or trimethoprim-sulphamethoxazole 86). Testosterone given for masculinising therapy can contribute to alopecia associated with tacrolimus, can cause acne which can be exacerbated with steroids and predispose to infectious vaginitis in individuals who have undergone female-to-male gender affirmation surgery. Testosterone can also increase erythropoiesis and contribute to the development of post-transplant erythrocytosis (18).

Transgender individuals have a high risk of psychiatric illnesses including anxiety, depression or substance abuse (85, 88). Mental health issues may contribute to medication non-adherence or declining appropriate therapy which may affect transplant outcomes. Thus involvement of mental health professionals in the transplant team is essential. Such illnesses can also be aggravated during periods of increased steroid dosages such as antirejection therapy. Changes in physical appearance with transplant medications may also lead to medication non-adherence such as the development of cushingoid body habitus with chronic steroid use, alopecia with tacrolimus or hirsutism with cyclosporine (89). Transgender kidney transplant patients require close psychosocial monitoring and social support alongside usual care for good transplant outcomes. A greater sensitivity and discretion on the part of the healthcare providers is required in managing their kidney disease.

## 6 Pregnancy and fertility after kidney transplantation

Fertility in CKD is low, owing to factors such as the dysfunctional interplay of gonadotropins and hypothalamic-pituitary axis and reduced renal clearance of prolactin among others (90). Hormonal changes often reverse with kidney transplantation and improve fertility (18). Metanalysis and systematic reviews have identified a higher live birth rate among transplant recipients but also noted a higher caesarean section rate, gestational diabetes, hypertension, pre-eclampsia, low birth weight and fetal loss (91, 92). Kidney transplant recipients may have pre-existing diabetes, hypertension and cardiovascular disease, or have advanced maternal age which may contribute to increased risk of complications (91). Certain transplant medications such as MMF are also teratogenic which can lead to increased spontaneous abortions and ear and facial fetal deformities and thus need substitution to azathioprine prior to planning pregnancy (92). Deshpande et al. reported an acute rejection rate of 4.2% during pregnancy among 2412 pregnant recipients (91). Studies assessing graft outcomes in pregnancy have been inconclusive and suffer from bias, with some showing greater acute and chronic graft loss in the first two years post-pregnancy (93) and others observing a comparable graft function with nulliparous controls, and no long-term effects on graft function, with a marginal higher impairment noticed in two years post-partum (94). Transplant recipients are usually counselled to avoid pregnancy in the first 2 years after transplant, and plan pregnancy only in the presence of stable graft function (serum creatinine <1.5mgdl) with an absence of significant proteinuria on stable pregnancy safe immunosuppression with no recent rejection event (18, 94). Safer methods of contraception in transplant recipients include progesterone-only pill or intramuscular depot injections, and intrauterine devices. Physical barrier methods such as condoms are useful in avoiding sexually transmitted diseases, however, are not very effective when used alone for contraception. Estrogen-containing oral contraceptive pills are associated with risks of venous thromboembolism or hypertension and are better avoided (92).

Men also have poor fertility in CKD, with low sperm counts and testosterone which improves following transplantation, with restoration of fertility. A significant proportion of men with CKD have erectile dysfunction, which often improves with kidney transplantation but may persist in 20-50% of patients, likely from medication effect, altered endocrine milieu, polyneuropathy, diffuse vascular disease and psychosocial problems which may impair quality of life (95).

## 7 Limitations of current evidence

The current information is based mainly on single-centre studies limited by small sample size, incomplete data about relevant variables and the influence of local centre practices. They may be biased by local social structure leading to gender disparity in access to healthcare and family support. There is a paucity of data from LMICs like India where most of the information comes from single-centre retrospective studies (16, 17). However, considering the findings are similar from different parts of the country, gender disparity seems to be widely prevalent.

The large multi-centric studies based on registry data (12, 19, 22, 46, 47, 69, 70) should also be interpreted with caution due to their retrospective design. At best, they report associations but cannot establish causation. There may be residual confounding as details of certain vital parameters like severity of comorbidities, cognitive function, dementia, medication compliance which may influence decisions regarding transplantation as well as socio-economic cultural factors which play an important role in gender inequality were not examined. Certain variables like obesity were captured at the time of diagnosis of ESRD (46) and not when the patient is waitlisted which may have led to misclassification. The gender is often assigned by the healthcare provider which may differ from the gender perception of the individual (22). The majority of these large, multi-centric or registry studies are from Western, high-income countries and the findings cannot be generalized to other regions, especially LMICs where women face significantly more social and financial barriers when seeking treatment. There is limited information on patient and healthcare providers’ perspectives about gender bias in transplantation (25, 29, 42) with the possibility of selection bias and lack of transferability of conclusions to other countries and regions. We need prospective studies with adequate global representation exploring socio-cultural, financial and psychological issues as well as medical factors contributing to gender inequality in access to transplantation.

Lastly the role of transgender and other gender identities has not been addressed in these studies.

## Conclusions

Sex and gender differences affect all aspects of kidney transplantation and need due consideration for improving patient and graft outcomes. Equitable access to Kidney Transplantation with gender neutrality should be the goal and LMICs should not be lagging behind the rest. More research is needed on differential aspects including access to transplants, immunosuppression protocols, genetic and hormonal influences. A greater sensitisation is required in the medical community regarding gender disparity in transplantation and efforts to dispel conscious or unconscious bias based on sex and gender should be made. Regular analysis of gender-specific national and regional data regarding kidney transplantation can help in increasing awareness and introspection. Social support groups can help in navigating the transplant process and help in removing psychosocial barriers to transplantation. Healthcare policies should be geared towards improving deceased organ donation, establishing regional and national paired kidney exchange programs and transplant registries. National and state-sponsored schemes for financial support and increasing community awareness would allow for equitable access to kidney transplantation among all sexes and genders. In the future, the development of precision medicine with the help of genomics and proteomics may help in optimising immunosuppression and follow-up protocols for all groups based on individual differences.

## Author contributions

AN: Writing – original draft, Writing – review & editing. SoB: Data curation, Writing – original draft, Writing – review & editing. ShB: Conceptualization, Data curation, Supervision, Visualization, Writing – original draft, Writing – review & editing.
